# Three-Dimensionally Conformal Porous Microstructured Fabrics *via* Breath Figures: A Nature-Inspired Approach for Novel Surface Modification of Textiles

**DOI:** 10.1038/s41598-017-02615-1

**Published:** 2017-05-24

**Authors:** Jianliang Gong, Bingang Xu, Xiaoming Tao

**Affiliations:** Nanotechnology Center, Institute of Textiles and Clothing, The Hong Kong Polytechnic University, Hung Hom, Kowloon, Hong Kong P. R. China

## Abstract

Breath figures (BFs) are a kind of water droplet arrays that can be formed by condensing aqueous vapor onto a cold surface, such as dewy phenomenon on a spider web. This study developed a BF-inspired approach for direct introduction of desired materials onto the textile surfaces with three-dimensionally conformal porous microstructures by the evaporation of solution-coated fabric under high humidity environment, which brings a brand-new kind of modified textiles, three-dimensionally conformal porous microstructured fabrics (CPMFs). Such kind of CPMFs can possess customized multifunctional properties of introduced materials, and meanwhile maintain the inherent properties and unique texture features of fabrics. This nature-inspired BF approach is robust and versatile for customized preparation of CPMFs based on different fabrics with different common polymers. Moreover, it is also feasible for one-step functionalization of CPMFs by the incorporation of nanoparticles (such as titanium dioxide nanoparticles, TiO_2_ NPs) into the porous microstructures during the BF process. Comparing to the sample modified without porous microstructures, the resultant TiO_2_ NPs-incorporated CPMFs show an obviously enhanced performance on photocatalytic degradation of pollutants under the same ultraviolet irradiation conditions.

## Introduction

Textiles are flexible and versatile materials consisting of natural or synthetic yarns (or fibers) that can be knitted, woven or nonwoven into designable network structures for a quite broad spectrum of applications. Owing to their light-weight, good breathability, flexibility and other excellent mechanical properties, textile-based materials and structures have recently attracted great attention in wearable electronics, such as triboelectric nanogenerators^1, 2^, supercapacitors and batteries^3–6^. In practical applications, textile materials often require further modification of textiles for the enhancement of some physicochemical properties or the introduction of certain advanced functions by direct coating (e.g. knife/roll coating, spraying, melting, deposition, laminating, etc.), surface treatment (e.g. corona discharge, flame treatment, plasma treatment, ultraviolet irradiation, wet chemical processing, graft polymerization, etc.) or their hybrid techniques^7–9^. These techniques are very valuable and have been well applied for textiles to improve or introduce some specific properties based on the different requirements of practical uses. However, it is noted that the introduction of solid coating layer usually conceals the inherent properties and unique texture features of fabrics, while the surface treatment often involves a complex or destructive process accompanying with the generation of surface defects. This inevitably results in the degradation of some other properties of textiles, such as flexibility, robustness and breathability.

Physical modification of the uneven fabric surfaces with three-dimensionally (3D) conformal formation of porous microstructures of introduced materials may provide an effective solution of synergizing the desired functions of materials and excellent properties of fabrics. Moreover, the specially designed porous structures can be further used as promising framework platform for customized incorporation of different functional components^10, 11^. The effective and popular strategies for the preparation of porous materials usually involve a templating process. And generally, any medium that can be removed by post-treatment can be explored for templating fabrication of porous microstructures, such as surfactants, colloidal particles, ionic liquids and biopolymers^12–17^. These similar templating techniques have been well studied for the fabrication of porous films or membranes on solid and planar surface, but they are not very suitable for the formation of 3D conformal porous microstructures contouring to the uneven surface of nonplanar substrates, particularly textiles. And such study is significantly less developed due to the considerable challenge in the balance of technologic feasibility, production efficiency and economic cost. To construct conformal porous microstructures on an irregular object, an indirection solution is to transfer the prepared porous films for coverage. However, this low-productivity strategy is only feasible for some nonplanar substrates with simple curved surface. For complex and flexible textile surface, some elaborate structure features will be concealed, and cracks will occur easily during the transferring process due to the stress concentration or other causes.

In this work, we present a unique strategy of using easily-available and nontoxic breath figures (BFs) as template medium for straightforward formation of 3D conformal honeycomb porous microstructured materials on fabrics, which brings a brand-new class of modified textiles, 3D conformal porous microstructured fabrics (CPMFs). Such CPMFs are promising supporters for the incorporation of customized components to meet different functional applications, such as incorporating titanium dioxide nanoparticles (TiO_2_ NPs) for photocatalytic degradation of pollutants.

## Results and Discussion

BFs are foggy or dewy phenomena of aqueous vapor condensing onto a cold surface^18, 19^. Recently, we have employed BFs as soft dynamic templates for producing the porous microstructures of diverse materials with adjustable size and shapes^20–22^, which possesses versatile, highly-effective, easily-scalable and non-destructive advantages for various applications^23–25^. Moreover, this promising technique requires no trivial work on the preparation and removal of templates because all the condensation, assembly and evaporation of BFs are spontaneous. Owing to the rapid and spontaneous characteristics, we consider that the BF strategy may also be feasible for the formation of porous microstructures on the flexible and nonplanar surface of textiles. Actually in nature, bright and pearl-like dewy drops hanging the silks of spider webs is a ubiquitous phenomenon under humid climate. Its main formation is largely ascribed to the driven forces caused by surface energy gradients and Laplace pressure difference^26^. Inspired by this, we exposed a copper/nickel coated polyester fabric (CNF) coated with a kind of chloroform (CHCl_3_) solution containing a silicon-containing graft copolymer poly(dimethylsiloxane)-*graft*-polyacrylates (PDMS-*g*-PAs) in a sealed saturated-humidity environment. CHCl_3_ is a highly volatile solvent with a low boiling temperature point of approximately 61.2 °C. And its vapor pressure is always higher than that of water in a wide temperature range^27^. The vapor pressure difference enables the continuous evaporation of solvent into the humidity environment, while the generated evaporative cooling leads to the condensation of water from its saturated vapor on the fabric coated solution. And because water possesses a much higher surface tension than CHCl_3_ (about 72.0 mN m^−1^ at 25 °C, which is much larger than CHCl_3_ of 27.1 mN m^−1^)^27^, this surface tension difference will always drive the condensed water to nucleate and grow on the solution surface in spherical shape with the continuous evaporation of solvent. After the complete evaporation of solvent and air drying, only polymers will be left on the fabric.

**Figure 1:**
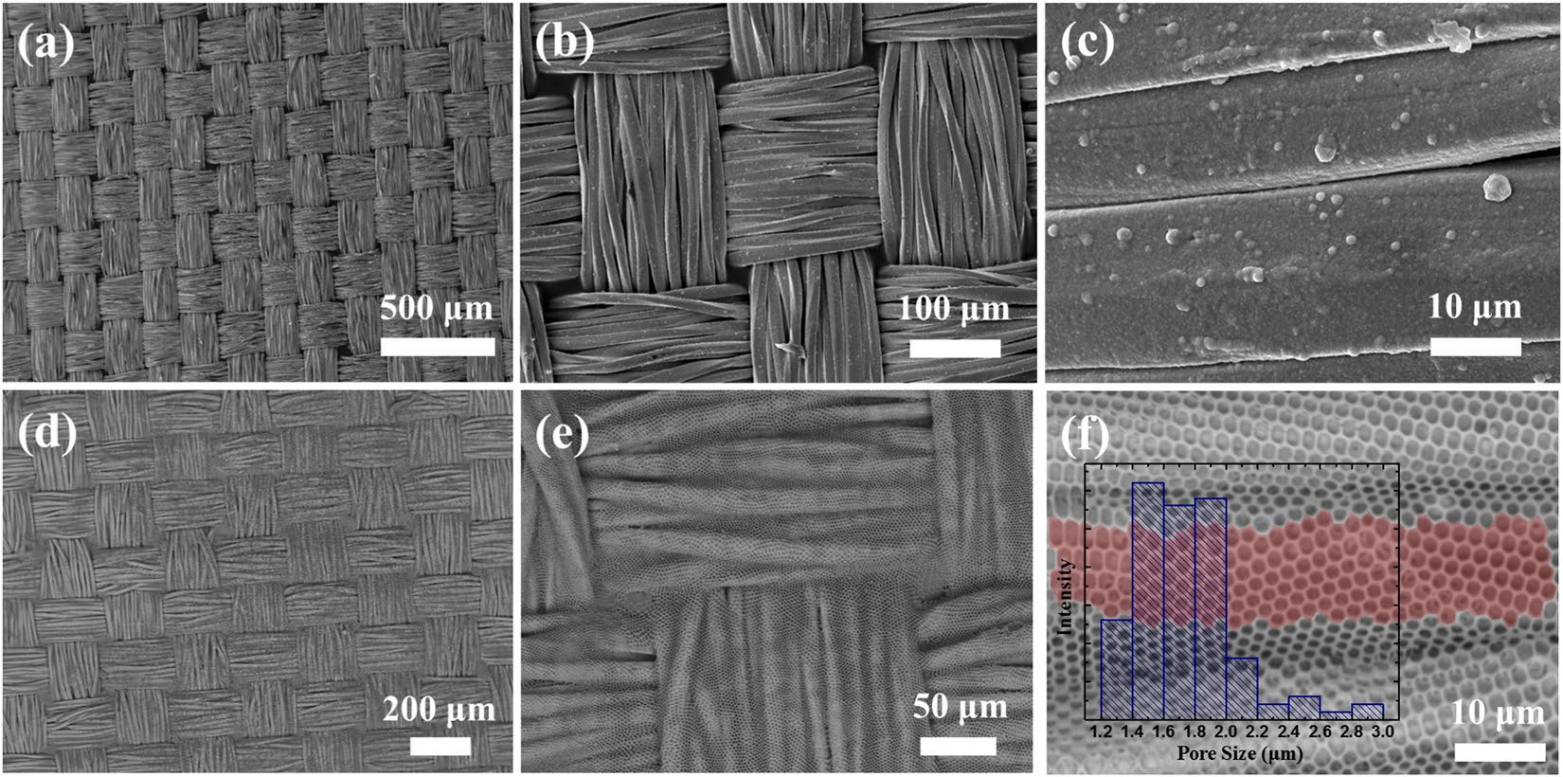
SEM images of CNF fabric (**a–c**) before and (**d–f**) after the conformal formation of 3D honeycomb porous microstructures *via* BFs. The concentration of casting solution was 15.0 mg mL^−1^. The inset is the pore size distribution of highlighted area.

The surface morphology of such polymer modified CNF from 15.0 mg mL^−1^ solution is highly identical with the texture feature of pristine CNF under the scanning electronic microscopy (SEM) at a low magnification (Fig. 1a and d). Figure 1b and c reveal that the pristine CNF is directly plain weaved by numerous fibers in the diameters of around 20 μm. After introduction of polymer, its fiber profiles in both longitudinal and latitudinal directions are still dinstict (Fig. 1e). Surprisingly at a closer view, they are actullay comformably covered with highly regular honeycomb porous microstructures, as demonstrated in Fig. 1f. Regardless of the complexity of fabric surface, the ordered porous microstructures well conform to the uneven fiber arrays without any cracks, which are believed to be the imprints of water microdroplets^28–30^. These results indicate that materials can be introduced as highly ordered and noncracking porous microstructures *via* BFs to modify fabric without concealing its unique texture features, thereby leading to the formation of well-constructed CPMF. Through the analysis of its relatively planar surface area (such as the area of Fig. 1f highlighted in transparent red), the pore sizes of obtained CPMF can be speculated to roughly range from 1.20 to 3.00 µm, as shown in the inset histogram of Fig. 1f. The average pore size is estimated at approximately 1.75 µm.

**Figure 2:**
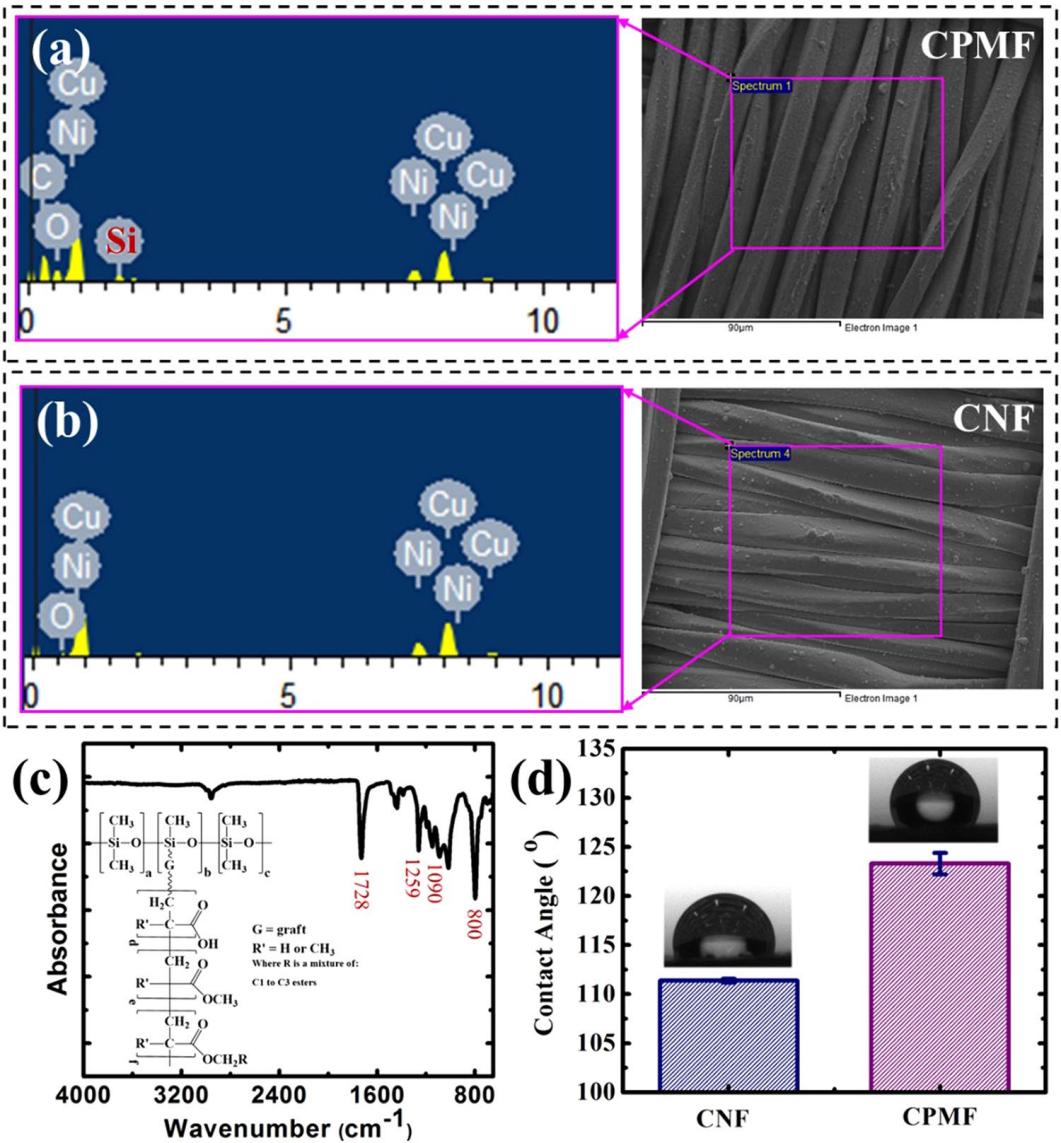
Typical EDX patterns of (**a**) CPMF and (**b**) CNF samples without gold coating, (**c**) FTIR spectrum and chemical structure of PDMS-*g*-PAs (inset), and (**d**) comparison of static contact angles of water droplets on CNF and CPMF. The insets of (**d**) are photographs of water droplets placed on CNF and CPMF, respectively.

The surface modification of CNF with PDMS-*g*-PAs by the BF approach was further confirmed by the analyses of energy dispersive X-ray spectrometer (EDX) and Fourier transformed infrared spectroscopy (FTIR). As shown in Fig. 2a, the EDX pattern of CPMF clearly demonstrates the existence of silicon element besides the characteristic signals of copper and nickle elements in the EDX pattern of CNF (Fig. 2b). And the former also possesses stronger signals of carbon and oxygen elements than the latter. When analyzing the powders scraped from CPMFs by FTIR, the characteristic peaks of stretching vibrations of C=O and Si-C are clearly observed at 1728 cm^−1^ and 1259 cm^−1^, respectively, while the absorption peaks at 1090 cm^−1^ and 800 cm^−1^ are attributed to the symmetric and asymmetric stretching vibrations of Si-O-Si (Fig. 2c). These analysis results are coincident to the chemical structure of used silicon-containg graft polymer PDMS-*g*-PAs (the inset of Fig. 2c), which well demonstrates that only polymers are left on the fabric surface after the BF process. Owing to the existence of low surface energy component and conformal porous microstructures, such PDMS-*g*-PAs modified CPMFs also show a more hydrophoboic surface than pristine CNF. As demonstrated in Fig. 2d and its insets, the static contact angle of water droplet on CPMF is 123.3 ± 1.1°, which is larger that of water droplet on unmodified CNF (111.4 ± 0.2°).

**Figure 3:**
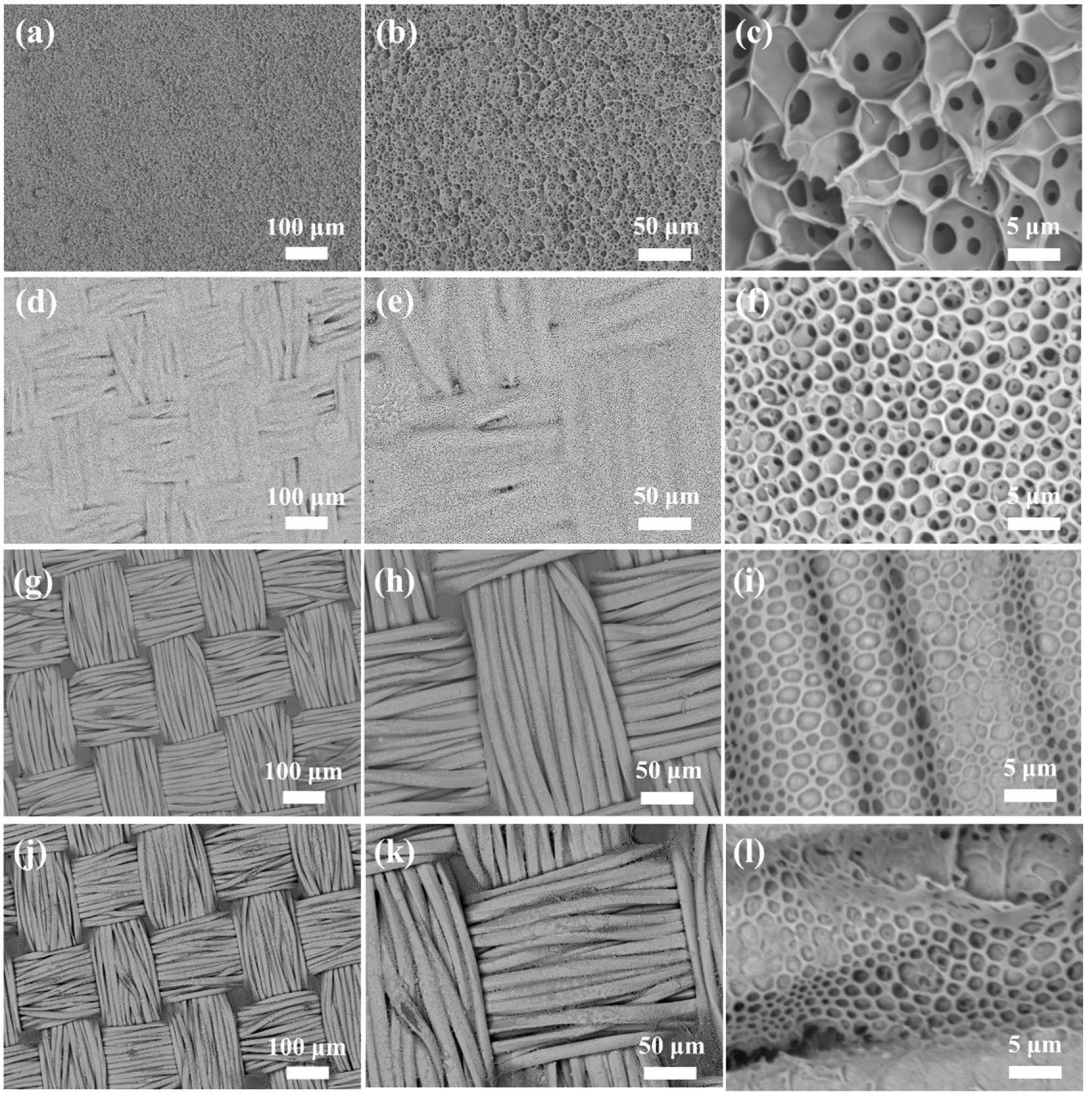
The influence of solution concentration on the formation of CPMFs: (**a–c**) 60.0 mg, (**d–f**) 30.0 mg mL^−1^, (**g–i**) 7.5 mg mL^−1^, and (**j–l**) 3.0 mg mL^−1.^

The influence of other initial concentrations of casting solutions (60.0 mg mL^−1^, 30.0 mg mL^−1^, 7.5 mg mL^−1^ and 3.0 mg mL^−1^) on the surface microstructures of CPMFs was shown in Fig. 3. Honeycomb porous microstructures were observed for all samples regardless of solution concentration. However, the texture feature of fabric was not obvious at high concentration (Fig. 3a–c), because there are emuch polymers to smooth the uneven surface. With the decrease of solution concentration, the fabric texture became observable (Fig. 3d), but some of its fiber profiles still cannot be distinguished (Fig. 3e and f). If the solution concentration was further decreased, the resultant CPMFs have a distinct view of fabric texture (Fig. 3g), but their honeycomb porous microstructures become less ordered and regular (Fig. 3h). At some areas, the porous microstructures are discontinuous (Fig. 3i). Further decrease of the solution concentration will aggravate this situation (Fig. 3l), although it can enable the surface morphology of CPMF more close to the texture feature of fabric (Fig. 3j and k).

**Figure 4:**
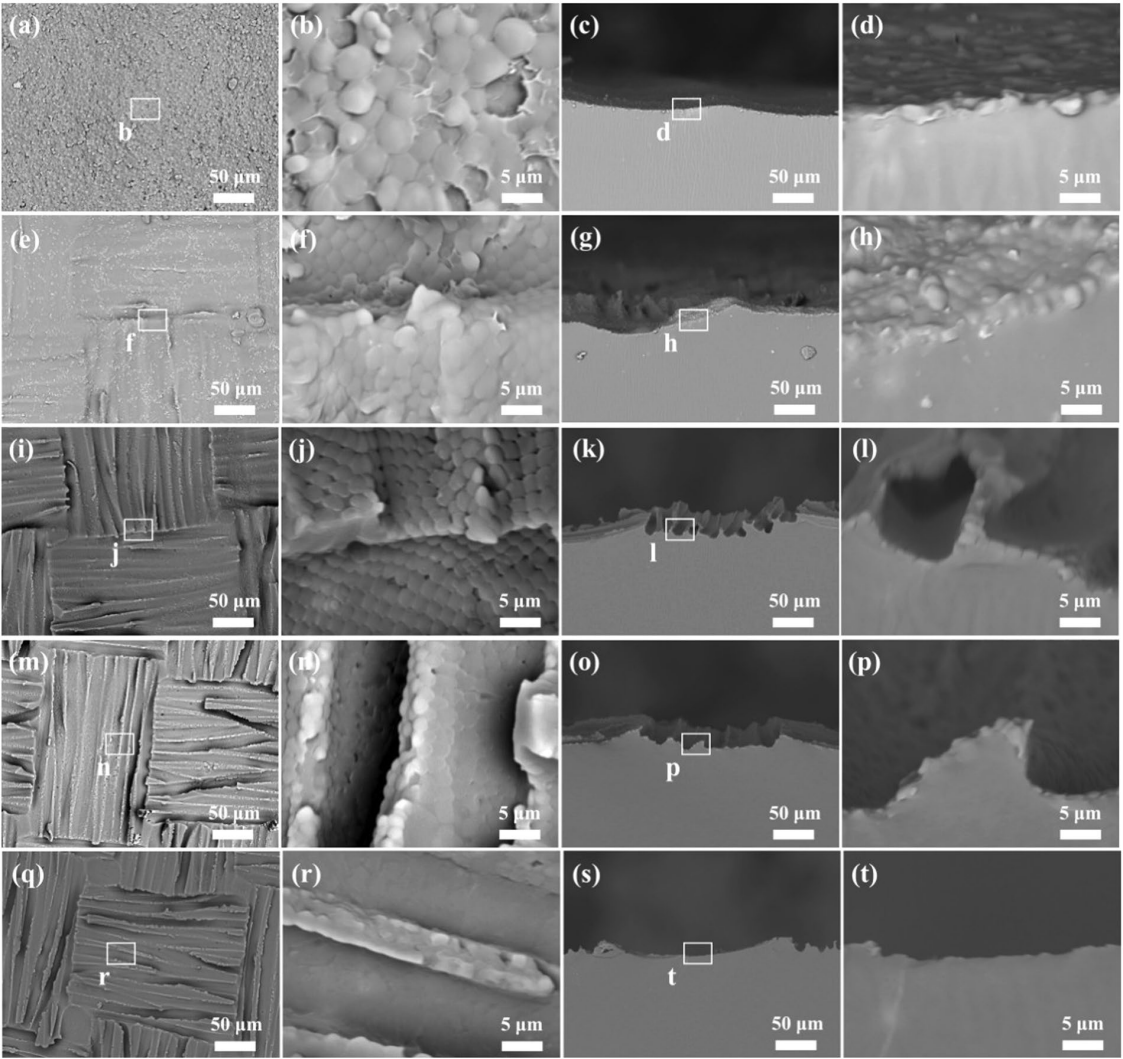
The positive PDMS replicas of CPMFs prepared from different solution concentration: (**a–d**) 60 mg, (**e–h**) 30 mg mL^−1^, (**i–l**) 15 mg mL^−1^, (**m–p**) 7.5 mg mL^−1^, and (**q–t**) 3.0 mg mL^−1^. The left and right two columns are the top views and cross-sectional views at different magnifications, respectively.

To further reveal the dimensional microstructures of CPMFs, their surface morphologies were replicated with polydimethylsiloxane (PDMS) films by molding synthesis. The top views in Fig. 4a and b indicate that the PDMS film templated from the CPMF prepared at high solution concentration has a convex surface decorated with micro lens arrays (MLAs). Its corresponding cross-sectional views in Fig. 4c and d confirm the formation of MLAs, and reveal that the surface profile is slightly curved. The MLA-decorated fabric texture of PDMS film at less high concentration becomes distinguishable (Fig. 4e and f), which displays a more curved surface profile (Fig. 4g and h). At a medium concentration, the resultant CPMF leads to the formation of distinct texture feature. And fiber-like channels are clearly observed in both meridional and parallel directions, which were well decorated with regularly beautiful MLAs (Fig. 4i and j). This benefits from the sharp height difference of uneven surface, which can be confirmed from their cross-sectional views (Fig. 4k and l). Such similar hierarchical surface features can also be obtained at a lower concentration (Fig. 4m–p).

However, when the CPMF from the solution with a very low concentration was used as negative mold, the fabric profile of resultant PDMS film is still explicit (Fig. 4q and s), but its MLA region is not obvious (Fig. 4t). And no regular and continuous MLAs can be observed from its top view (Fig. 4r). Only disordered micro lenses are found to intersperse in some local areas. These results demonstrate that the surface morphology evolution of PDMS replicas well coincides with the dimensional microstructure variation of CPMFs observed above, and indicate that the high concentration of casting solution will result in concealing the elaborate fabric texture features of CPMFs, while the low solution concentration will lead to the formation of CPMFs with less ordered and discontinuous honeycomb porous microstructures.

**Figure 5:**
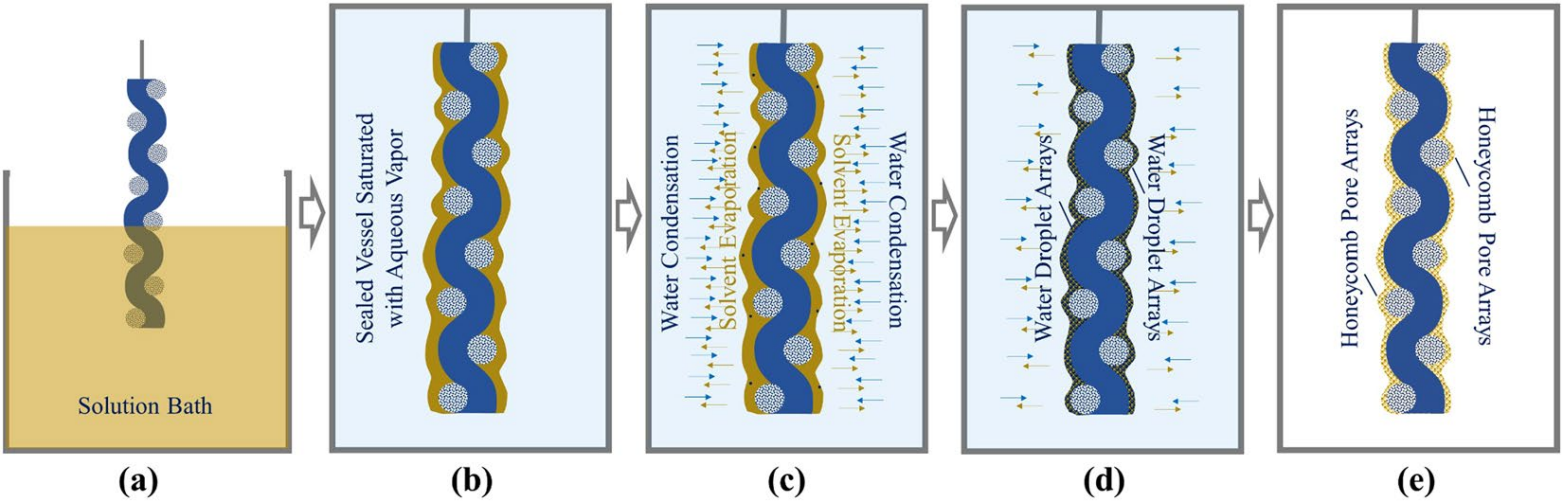
Schematic processes of preparing CPMFs *via* BFs, (**a**) introducing polymer solution onto fabric by dip coating, (**b**) transferring solution-coated fabric into a sealed vessel saturated with aqueous vapor quickly, (**c**) Condensing water onto the solution-coated fabric by evaporative cooling, (**d**) Retaining conformal water droplet arrays-containing polymer gels on fabric, and (**e**) Obtaining CPMFs after complete evaporation of water and solvent.

It is interesting to find the polymers conformably formed on uneven and untight fabric with honeycomb porous microstructures rather than simply filled in the sunken areas, because due to gravity it should be difficult for a flowing liquid to spread on nonplanar substratre homogeneously. The main reasons can be ascribed to the unique, rapid and spontaneous process of BF technique and the appropriate viscosity of polymer solution. During a typical process, the surface of a fabric is firstly covered with a layer of polymer solution by dip coating (Fig. 5a), and then quickly transferred to a closed environment saturated with aqueous vapor (Fig. 5b). The rapid evaporation of solvent will cool the solution surface, and lead to the condensation of water droplets by nucleation and growth (Fig. 5c). Owing to the high surface tension of water, the condensed water droplets will always tend to keep a spherical shape^20^. And they can further self-assemble into more ordered arrays driven by some complicated forces, such as Marangoni convection and thermocapillary effect^28, 30^. The condensation of nonsolvent water, increase of solution concentration and decrease of solution temperature will synergistically increase the viscosity of liquid system. This contributes to retaining the liquid film on the nonplanar surface. When the retaining time is long enough for the gelation of polymer solution, the sedimentation of polymers in sunken areas can be effectively avoided (Fig. 5d). After further evaporation of water and residual solvent by spontaneous drying in air, only conformal polymer layer with honeycomb porous microstructures will be left on the fabric (Fig. 5e). The formation of non-cracking honeycomb porous microstructures can be ascribed to the low glass transition temperature (*T*_g_) PDMS component (low to −125 °C), which endows PDMS-*g*-PAs microstructures with excellent elasticity to alleviate the concentrated stress caused by uneven surfaces and during the drying process.

**Figure 6:**
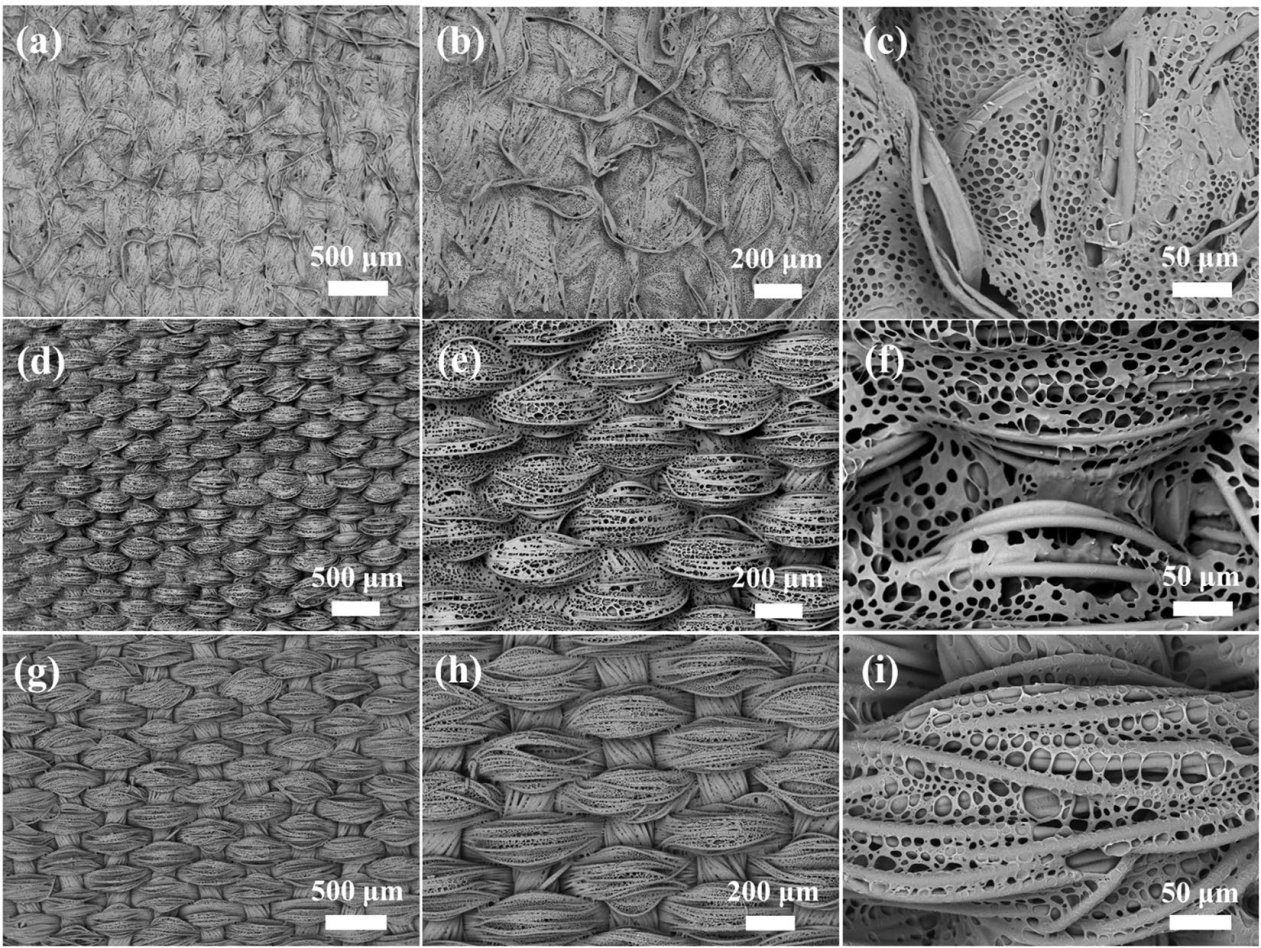
SEM images of CPMFs with different polymers on different fabrics: (**a–c**) PS/asphalt coated cotton fabric, (**d–f**) PS/asphalt coated polyester fabric, and (**g–i**) SBS coated polyester fabric.

This nature inspired BF strategy is also feasible and versatile for the preparation of CPMFs based on different fabrics with different common polymers, such as polystyrene (PS) and its block copolymer PS-*b*-polybutadiene-*b*-PS (SBS). Considering the brittle property of PS caused by its high *T*_g_ (about 100 °C), a semisolid asphalt was used as additive during the BF process to endow the finally obtained porous microstructures with better toughness. The resultant CPMFs based on cotton fabric with irregular texture and polyester fabric with regular texture were revealed by SEM at different magnifications, as shown in Fig. 6a–c,d–f, respectively. Polymers are found to be well introduced on the fabric and fiber surfaces with conformal honeycomb porous microstructures meanwhile maintaining the unique texture features of fabrics. SBS is a commercially available thermoplastic elastomer, which was also directly used for the preparation of CPMFs without any additives. SEM images at different magnifications in Fig. 6g–i show similar conformal honeycomb porous microstructures on polyester fabric substrate. These results indicate that CPMFs can be prepared with desired materials and fabrics to meet different requirements of practical uses.

**Figure 7:**
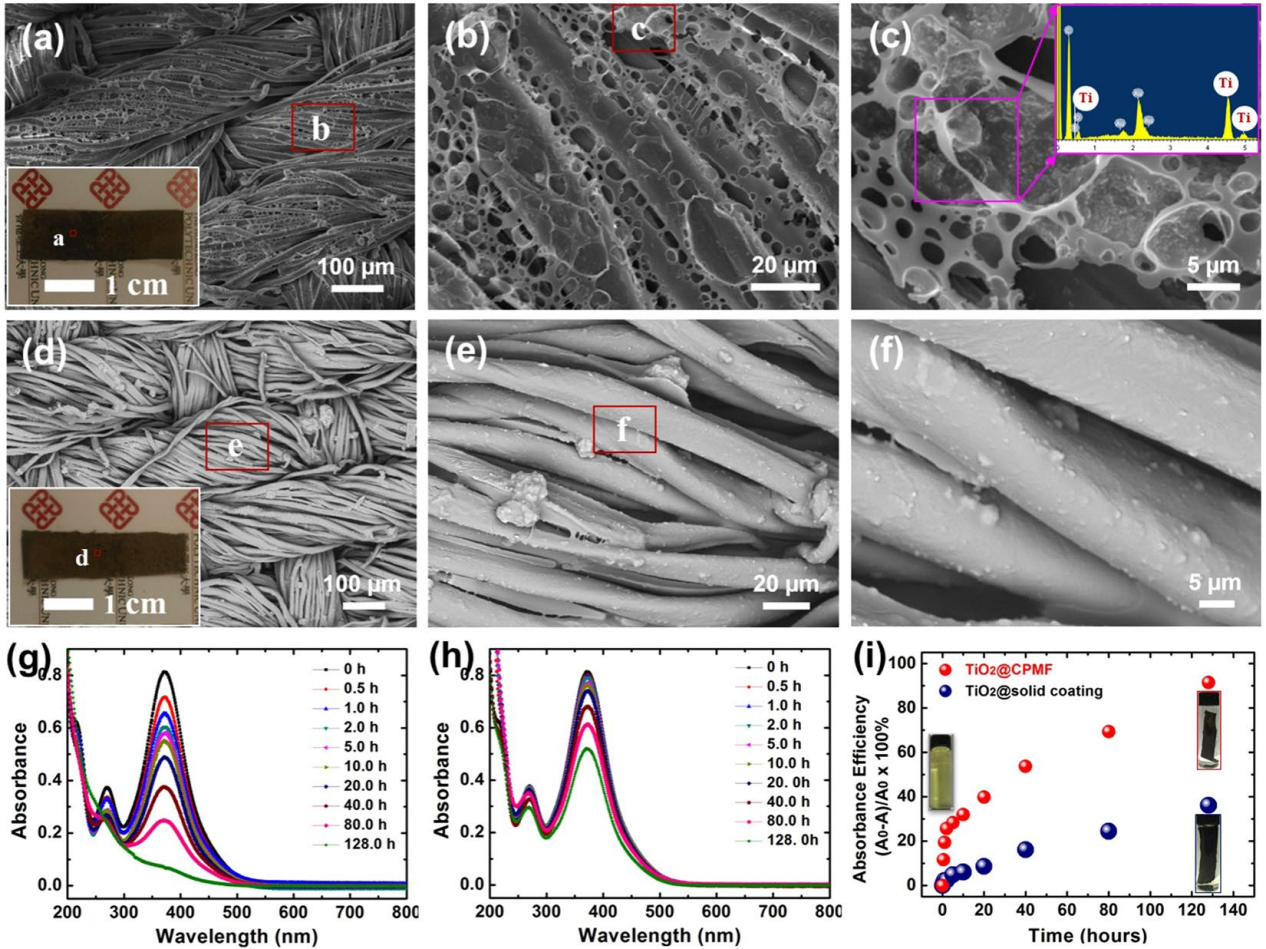
SEM images of (**a–c**) TiO_2_@CPMF and (**d–f**) TiO_2_@solid coated fabric at different magnifications. UV-visible spectral changes of MO1 aqueous solution (5 × 10^−5^ M) in the presence of (**g**) TiO_2_@CPMF and (**h**) TiO_2_@solid coated fabric with the exposure time of 365 nm UV irradiation. (**i**) The plot of removal efficiency of MO1 against time by different fabric-based samples. The insets of (**a**) and (**d**) were the photographs of 1 cm × 4 cm TiO_2_@CPMF and TiO_2_@solid coated fabric, respectively. The insets of (**i**) were the photographs of as-prepared MO1 aqueous solution and the MO1 aqueous solutions filled with different modified fabrics after 128 h UV irradiation time, respectively.

Porous structures are good platforms for loading different functional components because they can provide a larger specific surface area and alleviate the embedding of loaded components in the solid matrix. Another very encouraging advantage of this study is that desired nanoparticles can be directly incorporated into the porous microstructures for one-step fabrication of multifunctional CPMFs. Taking the fabrication of TiO_2_ NPs-incorporated CPMFs (TiO_2_@CPMFs) as an example, a given amount of TiO_2_ NPs was firstly dispersed in the PS/asphalt solution, and then introduced on the surface of polyester fabric by the same BF process stated above. Figure 7a and b show that TiO_2_@CPMFs have a similar surface features of CPMFs without the addition of nanoparticles. This indicates that the incorporation of TiO_2_ NPs has little influence on the formation of conformal porous microstructures on fabric. When taking a close view of porous microstructures, some pore walls are found to be decorated with particulate nanocomponents (Fig. 7c). They are further confirmed as TiO_2_ NPs by EDX (inset of Fig. 7c). The decoration of TiO_2_ NPs on pore walls can be ascribed to the famous Pickering emulsion effect^31^, which prefers driving nanoparticles to be selectively absorbed at the water/solution interface for the stabilization of water droplets during the BF process^32^. When the same casting solution was coated on the fabric by conventional method in ambient conditions, no porous microstructures are observed on the surface of fabric (Fig. 7d and e). The introduced nanoparticles are found to aggregate and adhere to the fiber together with polymers, as shown in Fig. 7f.

Benefiting from the photocatalytic properties of TiO_2_, the hybrid TiO_2_@CPMF with hierarchical porous structures can be further applied for the degradation of organic pollutants in water. To evaluate its photocatalytic performance, an azo dye, mordant orange 1 (MO1), was used as the model pollutant. The aqueous solution of MO1 displays a bright yellow at 5 × 10^−5^ M (inset of Fig. 7i). Figure 7g shows the UV-visible absorption spectra of MO1 aqueous solution treated by TiO_2_@CPMF under 365 nm UV lamp with different time. At 0 h, MO1 shows a strong absorption band at 371 nm. With the increase of UV exposure time, the typical band shows an obvious decrease trend. After 128 h, the absorbance decreased by about 91.5%. And the yellow solution becomes colorless (inset of Fig. 7i), reflecting that most of MO1 was degraded. By contrast, when TiO_2_/polymer coated fabric sample without porous microstructure (TiO_2_@solid coating) was immersed in MO1 solution with the same initial concentration for 128 h UV exposure, the solution color becomes light yellow (inset of Fig. 7i), but the declination of band intensity is only about 36.1% (Fig. 7h). The removal efficiency of MO1 by the fabric-based samples with different microstructures was further plotted against time, as shown in Fig. 7i. It clearly demonstrates that TiO_2_@CPMF possesses a quicker and stronger capacity of removing pollutants than the control sample without porous microstructures. The reasons for the low efficiency of control sample can be ascribed to the aggregation of TiO_2_ NPs and their embedment in the solid coating on fabric, which well indicates the advantageous porous microstructures of CPMFs for loading functional nanoparticles.

## Conclusion

In conclusion, an effective, straightforward and nondestructive strategy has been developed and demonstrated for the surface modification of fabrics with conformal porous microstructures of materials *via* BFs. Through the diversified combinations of fabrics and polymers, this BF-inspired strategy shows robust and versatile advantages for the preparation of CPMFs with customized properties. Moreover, it also has a great potential for direct incorporation of desired nanocomponents into porous microstructures for one-step fabrication of functional CPMFs with enhanced performance. This study may pave the way to a brand-new class of modified textiles that can possess customized multifunctional properties of introduced materials, inherent excellent properties (such as breathability and flexibility) and unique texture features of fabrics, which is believed to be promising materials for a wide spectrum of flexible and wearable applications.

## Methods

### Materials

The silicon-containing graft copolymer PDMS-*g*-PAs (*M*_n_ ~26,000, dimethylsiloxane composition, ~80 wt.%) was purchased from Sigma-Aldrich, Ltd. PDMS precursors were purchased from Momentive Performance Materials Japan LLC. CNF was brought from 3 M Corp. CHCl_3_ (anhydrous, ≥ 99.9%) was also obtained from Sigma-Aldrich Chemical Company. All materials were used as received without further purification.

### Preparation of CPMFs *via* BFs

A typical BF process for the preparation of CPMFs was operated in a sealed glass bottle with a cap at ambient temperature (23–25 °C). An approximately saturated humidity (the relative humidity is 99%) in the vessel was achieved by adding a small amount of distilled water into the bottle beforehand. Polymer solutions with concentration ranging from 3.0 to 60 mg mL^−1^ were prepared by dissolving PDMS-*g*-PAs in CHCl_3_. The well-tailored CNF was dipped into the prepared solution for coating, and then quickly moved to the sealed vessel for evaporation. After the evaporation of solvent, the sample was taken out for spontaneous air drying. To prepared TiO_2_@CPMFs, anatase TiO_2_ NPs were blended into the PS/asphalt solution according to the TiO_2_/polymer weight ratio of 1: 9. After ultrasonication and shaking for over ten minutes, the well-dispersed mixture was then immediately introduced on the polyester fabric by the same BF procedures.

### Molding synthesis of MLAs with CPMFs as templates

The as-prepared CPMFs *via* BFs acted as a negative mold. PDMS precursors were homogeneously mixed and then cast on the CPMF mold according to the mass-to-surface-area ratio of 0.048 g cm^−2^ by a doctor-blading method. Then it was shifted to a vacuum oven for degassing at room temperature to obtain PDMS liquid film without air bubbles. After the liquid film becoming transparent, the temperature was increased to 80 °C for cross-linking reaction, which was held for three hours. After cross-linkage, the PDMS replicas were directly peeled off from the CPMF molds carefully.

### Photocatalytic degradation of organic pollutant in water

Polyester fabric-based TiO_2_@CPMFs prepared above were tailored into 1 cm × 4 cm strip and immersed into a 10-mL bottle filled with MO1 aqueous solution. The aqueous MO1 solution was fixed at the concentration of 5 × 10^−5^ M. By contrast, the same size fabric sample with TiO_2_@solid coating was also immersed into the bottled MO1 solution. The control sample was prepared by the same BF procedures but in a dry ambient environment. The photochemical degradation process was performed by placing the bottles in a chamber within two UV light tubes. Each light tube generated UV emissions at a wavelength of 365 nm and power of 18 W. The distance between the UV source and fabric was 10 cm. A digital camera and an ultraviolet/visible (UV/vis) spectrometer were employed to monitor the degradation process of MO1 with time respectively.

### Characterization

CPMFs and PDMS replicas were coated with a thin layer of gold (around 2 nm) for imaging by a scanning electron microscope (SEM, JEOL Model JSM-6490). A 10 keV electron beam was used for the observation at a working distance of 10 mm. The elemental analysis of samples was conducted with SEM equipped an EDX that analyzes elements down to boron. The fracture surfaces of PDMS replicas were obtained by rapidly tearing for the cross-sectional views by SEM. The static contact angles of water were measured on the pristine and modified fabrics with a JGW-360C optical contact angle system at room temperature. The powders scraped from CPMFs were collected for FTIR analysis by using a Perkin-Elmer spectrum-100 FTIR spectrometer (Perkin-Elmer Instruments) with a universal ATR (attenuated total reflectance) sampling accessory. The sample was scanned 16 times and obtained a FTIR spectrum recorded in the range of 650–4000 cm^−1^ with the resolution of 2 cm^−1^. UV/vis spectra were recorded on a Lambda 18 UV/VIS Spectrometer.
